# Exact and numerical optical soliton solutions of the fractional quadratic–cubic nonlinear Schrödinger equation in conformable derivative framework

**DOI:** 10.1038/s41598-026-43272-7

**Published:** 2026-03-26

**Authors:** Aamna Amer, Emad K. Jaradat, Hamood Ur Rehman, Yakup Yildirim, Sharif Abu Alrub, M. S. Ateto

**Affiliations:** 1 Department of Mathematics, University of Okara, Okara, Pakistan; 2 Department of Physics, Faculty of Science, Imam Mohammad Ibn Saud Islamic University (IMSIU), 11623 Riyadh, Saudi Arabia; 3 Department of Computer Engineering, Biruni University, 34010 Istanbul, Turkey; 4 Mathematics Department, Faculty of Science, South Valley University, Qena, Egypt

**Keywords:** Quadratic–cubic nonlinear Schrödinger equation, Optical solitons, Conformable derivative, Fractional differential equations, Differential transform method, Nonlinear equations, Mathematics and computing, Optics and photonics, Physics

## Abstract

This article introduces new optical soliton solutions for a fractional version of the quadratic–cubic nonlinear Schrödinger equation that describes the transmission of optical pulses in fiber optic systems with superfast fibers. The solutions are obtained using the modified Sardar sub-equation method and the $(\frac{1}{\varphi (\zeta )},\frac{\varphi ^{'}(\zeta )}{\varphi (\zeta )})$ method. The model is transformed into a non-linear fractional partial differential equation with a non-integer order using the conformable derivative, which is an efficient fractional derivative. The proposed methods work by adding a new variable to the equation to convert its form into a non-linear equation with ordinary derivatives. A comparative analysis of the solutions is carried out, and the effect of varying the fractional parameter values on the behavior of the obtained solutions is investigated. The paper’s novelty lies in the fact that no previous articles have identified the new solutions obtained through the application of these two analytical methods. The acquired solutions include kink, bright, periodic, dark-bright, singular and dark-singular wave solutions, which are illustrated using several 3D and 2D graphs. Furthermore, to effectively validates the exactness of analytical solutions with an elevated precision, a numerical method known as differential transform method is carried out. The adopted approaches demonstrate notable performance and are suitable for solving other nonlinear partial differential equations that arise in the natural sciences.

## Introduction

The existence of nonlinear phenomenons in the natural world has piqued the interest of scholars. The analysis of the behavior of natural events has primarily relied on the use of partial differential equations^1–3^. To determine the exact and approximate traveling wave solutions, particular research is conducted based on distinct models. Considering that the physical properties of each model are crucial to its applications, these solutions aid in the discovery of novel characteristics of these models. These applications have far-reaching implications in numerous disciplines, including chemistry, engineering, biology, and nuclear and atomic sciences^4–7^. There are two distinct kinds of solutions to the non-linear partial differential equation (NLPDE): integer or fractional^8–12^. Recently, there has been discussion on the second kind of NLPDE. This class of NLPDEs is explored and studied via numerous fractional definitions including the fractional Riemann-Liouville derivatives, the conformable fractional derivative, Caputo-Fabrizio derivative, Caputo fractional derivative, and many recently discovered fractional derivatives. These definitions are employed to convert the NLPDE problems with fractional order into an ordinary differential equation (ODE) with integer order^13–18^. Various efficient and accurate methods have been presented to construct the exact solutions of the nonlinear Schrödinger equation (NLSE) up to now, including, extended hyperbolic function method^19^, the tanh-function expansion and its various modifications^20^, the $(G'/G)$-expansion method^21^, the variational iteration technique^22^, the extended rational sine-cosine and rational sinh-cosh method^23^, the modified Khater method^24^, the sine-Gordon expansion (SGE) method^25–29^, the first integral method^30^, the Sardar sub-equation method^31^, the extended Tanh-Coth method^32^, the Jacobi elliptic function (JEF) method^33^, and much more^34–36^.

Schrödinger equation is one of the models with a large number of formulas; numerous mathematicians and physicists, have dedicated their efforts to finding closed-form solutions for this important model. The model is classified as a member of the family of PDEs that analyse the behaviour of quantum mechanical systems by studying their wave function. Gao *et* *al*. in 2015 examined NLSE for optical solitons using $(\frac{m+G'}{G})$ expansion method and the $e^\phi$ expansion technique^37^. Fujioka *et* *al*. in 2011, explored the chaotic solitons of the model^38^. Yokus *et* *al*. examined the modified $(\frac{I}{G'})$ expansion scheme and Kudrayashov technique to investigate hyperbolic complex solitary waves in optical fibers and plasmas^39^. In 2006, Galaktionov and Svirshchevskii utilized various methods to discover exact and solitary traveling wave solutions for this model^40^. A research on a sub-eq scheme is conducted by Cheema *et* *al*. to investigate the NLSE for wave solutions^41^. Kaplan *et* *al*. have examined the $(\frac{I}{G}, \frac{G'}{G})$ expansion method and $(\frac{I}{G'})$ expansion methodology for determining exact solutions of the (1+1) dimensional NLSE^42^. By employing the method of undetermined coefficients Triki *et* *al*. in 2017, discovered optical soliton solutions of our model^43^. Li *et* *al*. have conducted research on (2+1) dimensional time-fractional NLSE via $(\frac{G'}{G})$ expansion technique^44^.

Over the past half-century, these researchers have successfully developed powerful methods to derive solitary traveling wave solutions and closed-form solutions for NLPDEs, which are considered novel by many^45–49^.

Despite the use of various analytical methods for quadratic–cubic and related nonlinear Schrödinger models, the role of conformable fractional order terms in the diversity and structural transitions of soliton solutions has not been adequately investigated. In particular, prior studies reported families of bright and dark solitons and certain periodic solutions, but they did not produce the combination forms like, dark–bright and dark–singular and some of the singular/rational closed forms obtained here. In this work, the modified Sardar sub-equation method (MSSM) and the $(\frac{1}{\varphi (\zeta )},\frac{\varphi ^{'}(\zeta )}{\varphi (\zeta )})$ method are used as the tools for exploring the complex solution morphologies made possible by the fractional parameters. These methods are one of the most effective approaches for many types of solitary wave solutions in addition to being a natural extension of many other approaches in this field. Furthermore, the accuracy of the acquired solutions is verified by the numerical validation utilizing the Differential Transform Method (DTM). Researchers interested in the physical attributes of these models can gain further insight into their properties and prospective applications due to the plethora of available solutions.

The motivation of this study is to elucidate the intricate dynamics of optical phenomena, with a specific focus on the QC-FNLSE in highly nonlinear media. The growing importance of the QC-FNLSE, especially in light of developments of propagation of optical pluses in optical fibers, highlights the relevance of our research. This study enhances our comprehension of the behaviour of the governed equation and offers novel insights by employing analytic methods that have not been previously utilized on QC-FNLSE.

The space-time fractional QC-FNLSE can be expressed as;

1 $$\begin{aligned} \iota D_t^\gamma \psi + \pi D_x^{2\gamma }\psi -\eta \psi |\psi |+\lambda \psi |\psi |^2=0. \end{aligned}$$

where $\iota =\sqrt{-1}$, and the real-valued constant $\pi$ indicates group velocity dispersion (GVD), which governs pulse broadening in optical fibers. Also $\psi =\psi (x,t)$ is the complex envelope function, describes the evolution of the optical field, with the independent variables *t* and *x* representing the temporal and spatial variables, respectively. The coefficients $\eta$ and $\lambda$ represent the quadratic and cubic nonlinear responses of the medium, respectively, taking into account the asymmetric wave interactions and Kerr-type self-phase modulation, respectively. The combined effect of these coefficients allows for more complex soliton dynamics than would be possible in the cubic nonlinear model. The operators $D^\gamma _t$ and $D^{2\gamma }_x$ represent the conformable fractional derivatives with respect to time and space, respectively, with the order $0<\gamma <1$, where the fractional parameter $\gamma$ captures the memory and nonlocal dispersion properties related to complex or designed optical media. As a result, the values of $\gamma$ represent a physically significant control parameter for adjusting pulse localization, amplitude, and shape evolution.

In this work, however, we concern on solving the system with a conformable fractional order derivative of the QC-FNLSE utilizing the complex wave transformation along with the MSSM and the $(\frac{1}{\varphi (\zeta )},\frac{\varphi ^{'}(\zeta )}{\varphi (\zeta )})$ method to extract the exact solutions^50^. By transforming the fractional PDE into an ODE through an appropriate complex fractional travelling-wave transformation, we derive new families of bright, dark, periodic, kink-type, singular, and mixed soliton solutions. We then illustrate how variations in the fractional order $\gamma$ affect the amplitude, width, and localization properties of these solutions, offering insight into the role of memory effects in fractional optical media. Additionally, quantitative numerical validation utilizing DTM, along with convergence analysis, is conducted.

The approach of this paper is systematized as; Sect. 2 introduces some results and definitions of conformable fractional derivative. In Sect. 3, brief explication of methods is introduced. In Sect. 4, we perform a suitable wave transformation to obtain the wave equation of the QC-FNLSE. We utilized the proposed methods to construct many kinds of exact solutions in Sect. 5. Section 6 gives the description of the anticipated DTM scheme. Discussions of some obtained solutions are discussed in Sect. 7. Section 8, finally winds up with some brief conclusions.

## Conformable fractional derivative

A generalization of the derivative of integer-order is the fractional derivative. A fractional derivative is a more effective way to describe engineering and physical problems. Not only it is theoretically significant, but also it has many practical applications. Various operators in fractional calculus, such as Atangana-Baleanu derivatives, the Riemann Liouville, the Caputo Fabrizio, and the Beta-time Derivative, have been developed for the study of fractional NLPDEs, in contrast to the unique formulations for integer-order derivatives. The conformable derivative offers a local fractional derivative concept that retains many standard derivative properties like linearity and the product rule, facilitating easier theoretical and computational treatments. The conformable derivatives are simpler and more effective to transform the considered equation into ordinary differential equations (ODE) by converting the wave variable. Therefore, it aids us in comprehending the characteristics of systems that may be observed.

Below, we provide the precise definition and certain characteristics of the conformable fractional derivative with order $\gamma$:

### Definition

Let $j:[0,\infty )\rightarrow R$ the conformable fractional derivative of *j* of order $\gamma$ is given as^51,52^

$$\begin{aligned} D_t^\gamma j(t)=\lim _{\delta \rightarrow 0} \frac{j(t+\delta t^{1-\gamma })-j(t)}{\delta }, \end{aligned}$$

for all $t>0$ and $0<\gamma \le 1$. If *j* is $\gamma$-differentiable in some interval $(0,\gamma )$, where $\gamma >0$ and $\lim _{t\rightarrow 0^+}D_t^\gamma j(t)$ exists, then define $D_t^\gamma j(0)=\lim _{t\rightarrow 0^+}D_t^\gamma j(t)$.

### Theorem 1

Consider $\gamma \in (0,1]$ and the functions *j* and *k* are $\gamma$-differentiable for all $t>0$ and $b_1$ and $b_2$ are all real constants. Then few results of the conformable fractional derivative are as;

(i) $$D_t^\gamma (b_1 j+b_2k)=b_1D_t^\gamma (j)+b_2D_t^\gamma (k).$$

(ii) $$D_t^\gamma (t^p)=pt^{t-\gamma },~\forall p\in R.$$

(iii) $D_t^\gamma (\Theta )=0,$ where $\Theta$ is a constant.

(iv) $$D_t^\gamma (jk)=jD_t^\gamma (k)+kD_t^\gamma (j).$$

(v) $$D_t^\gamma (\frac{j}{k})=\frac{k D_t^\gamma (j)-j D_t^\gamma k}{k^2}.$$

(vi) If *j* is differentiable then, $D_t^\gamma j(t)=t^{1-\gamma } \frac{dj}{dt}$.

### Theorem 2

Suppose $j:[0,\infty )\rightarrow R$ is differentiable and also $\gamma$-differentiable function with $\gamma \in (0,1].$ let *k* be a differentiable function defined in the range of *j*, then we have^53^;

$$\begin{aligned} D_t^\gamma (j\circ k)(t)=t^{1-\gamma }k'(t)j'(k(t)). \end{aligned}$$

## Explication of the method

Here we provide a concise overview of the methods and utilize them to obtain various precise solutions for Eq. (1).

Consider the following NLPDE involving $\nu (t,x_1,x_2,...,x_n)$ and its various fractional derivatives

2 $$\begin{aligned} G(\nu ,D_t^\gamma \nu ,D_{tt}^{2\gamma }\nu ,...,D_{x_1}^\gamma \nu ,D_{x_1}^{2\gamma } \nu ,...,D_{x_n}^\gamma \nu ,D_{x_n}^{2\gamma }\nu ,...,D_{x_1} t^\gamma \nu ,...)=0, \end{aligned}$$

where $0<\gamma \le 1$. Using the wave variable transformation

3 $$\begin{aligned} \nu =\nu (t,x_1,x_2,...,x_n)={\mathcal {V}}(\varepsilon ),~ \varepsilon =x_1+x_2+...+x_n-\frac{c}{\gamma }t^{\gamma }, \end{aligned}$$

where *c* is velocity. Eq. (2) reduces to the following ODE after the substitution of Eq. (3):

4 $$\begin{aligned} H({\mathcal {V}},{\mathcal {V}}',{\mathcal {V}}'',{\mathcal {V}}''',...)=0, \end{aligned}$$

where $'$ represents derivative w.r.t $\varepsilon$. Integrating Eq. (4) as many times as possible. For the purpose of studying soliton solutions, we may ignore the constant of integration. Here are the key procedures of the methods that have been proposed:

### The MSSM

The MSSM is an influential technique to determine the exact solutions to the non-linear evolution equations (NLEEs). By using this technique, a range of innovative traveling wave solutions of QC-FNLSE are generated in this article. The outcomes produced through this method are distinct and unique as compared to previous methods.

It is believed that the solution of Eq. (4) as^54^:

5 $$\begin{aligned} \nu (\zeta )=\Upsilon _{0}+\sum _{r=1}^{\varsigma }\Upsilon _{r}\phi ^{r}(\zeta ),~~~~\Upsilon {\varsigma }\ne 0, \end{aligned}$$

where $\Upsilon _{r},~r={0, 1,..., \varsigma }$ are constants. By using homogeneous balance rule, the highest derivative in Eq. (4) and the nonlinear terms can be balanced to determine the integer $\varsigma$. Further, the function $\phi (\zeta )$ in Eq. (5) fulfils the subsequent equation:

6 $$\begin{aligned} (\phi '(\zeta ))^2=\varrho _{2}\phi ^4(\zeta )+\varrho _{1}\phi ^2(\zeta )+\varrho _{0}, \end{aligned}$$

where the constants are $\varrho _{0}$, $\varrho _{1}$, and $\varrho _{2}$. Now the general solutions for Eq. (6) with parameter $\rho$ are presented as below:

1. If $\varrho _{0}=0$, $\varrho _{1}>0$, and $\varrho _{2}\ne 0$, then

7 $$\begin{aligned} \phi _{1}^\pm (\zeta )= & \pm \sqrt{-\frac{\varrho _{1}}{\varrho _{2}}}~sech(\sqrt{\varrho _{1}}(\zeta +\rho )), \end{aligned}$$

8 $$\begin{aligned} \phi _{2}^\pm (\zeta )= & \pm \sqrt{\frac{\varrho _{1}}{\varrho _{2}}}~csch(\sqrt{\varrho _{1}}(\zeta +\rho )). \end{aligned}$$

2. For two constants $\beta _{1}$ and $\beta _{2}.$ Let $\varrho _{2}=\pm 4 \beta _{1}\beta _{2}$, $\varrho _{1}>0$, and $\varrho _{0}=0$, we get

9 $$\begin{aligned} \phi _{3}^\pm (\zeta )=\pm \frac{4\beta _{1}\sqrt{\varrho _{1}}}{(4\beta _{1}^2-\varrho _{2})~cosh(\sqrt{\varrho _{1}}(\zeta +\rho ))\pm (4\beta _{1}^2+\varrho _{2})~sinh(\sqrt{\varrho _{1}}(\zeta +\rho ))}. \end{aligned}$$

3. If $\varrho _{0}=\frac{\varrho _{1}^2}{4\varrho _{2}}$, $\varrho _{1}<0$, $\varrho _{2}>0$, with constants $A_1$ and $A_2,$ then

10 $$\begin{aligned} \phi _{4}^\pm (\zeta )= & \pm \sqrt{-\frac{\varrho _{1}}{2\varrho _{2}}}~tanh(\sqrt{-\frac{\varrho _{1}}{2}}(\zeta +\rho )), \end{aligned}$$

11 $$\begin{aligned} \phi _{5}^\pm (\zeta )= & \pm \sqrt{-\frac{\varrho _{1}}{2\varrho _{2}}}~coth(\sqrt{-\frac{\varrho _{1}}{2}}(\zeta +\rho )), \end{aligned}$$

12 $$\begin{aligned} \phi _{6}^\pm (\zeta )= & \pm \sqrt{-\frac{\varrho _{1}}{2\varrho _{2}}}~(tanh(\sqrt{-2\varrho _{1}}(\zeta +\rho ))\pm \iota ~sech(\sqrt{-2\varrho _{1}}(\zeta +\rho ))), \end{aligned}$$

13 $$\begin{aligned} \phi _{7}^\pm (\zeta )= & \pm \sqrt{-\frac{\varrho _{1}}{8\varrho _{2}}}~(tanh(\sqrt{-\frac{\varrho _{1}}{8}}(\zeta +\rho ))+ coth(\sqrt{-\frac{\varrho _{1}}{8}}(\zeta +\rho ))), \end{aligned}$$

14 $$\begin{aligned} \phi _{8}^\pm (\zeta )= & \pm \sqrt{-\frac{\varrho _{1}}{2\varrho _{2}}}\left( \frac{\pm \sqrt{A_1^2+A_2^2}-A_1 ~cosh(\sqrt{-2\varrho _{1}}(\zeta +\rho ))}{A_1 ~sinh(\sqrt{-2\varrho _{1}}(\zeta +\rho ))+A_2}\right) , \end{aligned}$$

15 $$\begin{aligned} \phi _{9}^\pm (\zeta )= & \pm \sqrt{-\frac{\varrho _{1}}{2\varrho _{2}}}\left( \frac{cosh(\sqrt{-2\varrho _{1}}(\zeta +\rho ))}{ sinh(\sqrt{-2\varrho _{1}}(\zeta +\rho ))\pm \iota }\right) . \end{aligned}$$

4. If $\varrho _{0}=0$, $\varrho _{1}<0$, and $\varrho _{2}\ne 0$, then

16 $$\begin{aligned} \phi _{10}^\pm (\zeta )= & \pm \sqrt{-\frac{\varrho _{1}}{\varrho _{2}}}~sec(\sqrt{-\varrho _{1}}(\zeta +\rho )), \end{aligned}$$

17 $$\begin{aligned} \phi _{11}^\pm (\zeta )= & \pm \sqrt{-\frac{\varrho _{1}}{\varrho _{2}}}~csc(\sqrt{-\varrho _{1}}(\zeta +\rho )). \end{aligned}$$

5. If $\varrho _{0}=\frac{\varrho _{1}^2}{4\varrho _{2}}$, $\varrho _{1}>0$, $\varrho _{2}>0$, and $A_1^2-A_2^2>0,$ then

18 $$\begin{aligned} \phi _{12}^\pm (\zeta )= & \pm \sqrt{\frac{\varrho _{1}}{2\varrho _{2}}}~tan(\sqrt{\frac{\varrho _{1}}{2}}(\zeta +\rho )), \end{aligned}$$

19 $$\begin{aligned} \phi _{13}^\pm (\zeta )= & \pm \sqrt{\frac{\varrho _{1}}{2\varrho _{2}}}~cot(\sqrt{\frac{\varrho _{1}}{2}}(\zeta +\rho )), \end{aligned}$$

20 $$\begin{aligned} \phi _{14}^\pm (\zeta )= & \pm \sqrt{\frac{\varrho _{1}}{2\varrho _{2}}}~(tan(\sqrt{2\varrho _{1}}(\zeta +\rho ))\pm sec(\sqrt{2\varrho _{1}}(\zeta +\rho ))), \end{aligned}$$

21 $$\begin{aligned} \phi _{15}^\pm (\zeta )= & \pm \sqrt{\frac{\varrho _{1}}{8\varrho _{2}}}~(tan(\sqrt{\frac{\varrho _{1}}{8}}(\zeta +\rho ))- cot(\sqrt{\frac{\varrho _{1}}{8}}(\zeta +\rho ))), \end{aligned}$$

22 $$\begin{aligned} \phi _{16}^\pm (\zeta )= & \pm \sqrt{\frac{\varrho _{1}}{2\varrho _{2}}}\left( \frac{\pm \sqrt{A_1^2-A_2^2}-A_1 ~cos(\sqrt{2\varrho _{1}}(\zeta +\rho ))}{A_1 ~sin(\sqrt{2\varrho _{1}}(\zeta +\rho ))+A_2}\right) , \end{aligned}$$

23 $$\begin{aligned} \phi _{17}^\pm (\zeta )= & \pm \sqrt{\frac{\varrho _{1}}{2\varrho _{2}}}\left( \frac{cos(\sqrt{2\varrho _{1}}(\zeta +\rho ))}{ sin(\sqrt{2\varrho _{1}}(\zeta +\rho ))\pm 1}\right) . \end{aligned}$$

6. If $$\varrho _{0}=0$$, $$\varrho _{1}>0$$, then

24 $$\begin{aligned} \phi _{18}^\pm (\zeta )= & \frac{4\varrho _{1}e^{\pm \sqrt{\varrho _{1}}(\zeta +\rho )}}{e^{\pm 2 \sqrt{\varrho _{1}}(\zeta +\rho )}-4\varrho _{1} \varrho _{2}}, \end{aligned}$$

25 $$\begin{aligned} \phi _{19}^\pm (\zeta )= & \frac{\pm 4\varrho _{1}e^{\pm \sqrt{\varrho _{1}}(\zeta +\rho )}}{1-4\varrho _{1} \varrho _{2}e^{\pm 2 \sqrt{\varrho _{1}}(\zeta +\rho )}}. \end{aligned}$$

7. If $\varrho _{0}=\varrho _{1}=0$, and $\varrho _{2}>0$ then

26 $$\begin{aligned} \phi _{20}^\pm (\zeta )=\pm \frac{1}{\sqrt{\varrho _{2}}(\zeta +\rho )}. \end{aligned}$$

8. If $\varrho _{0}=\varrho _{1}=0$, and $\varrho _{2}<0$ then

27 $$\begin{aligned} \phi _{21}^\pm (\zeta )=\pm \frac{\iota }{\sqrt{-\varrho _{2}}(\zeta +\rho )}. \end{aligned}$$

Substituting Eqs. (5) and (6) into (4), afterwards, a set of algebraic equations with unknown variables is produced by collecting and equating all of the coefficients of $\phi ^{r}(\zeta )$ to zero. In the end, we employ Eqs. (7)-(27) to attain the exact solutions of Eq. (2) after solving the relevant set of equations and inserting these variables into Eq. (5).

### Description of the $(\frac{1}{\varphi (\zeta )},\frac{\varphi ^{'}(\zeta )}{\varphi (\zeta )})$ method

Using this method^55,56^, we can deduce that the solution to Eq. (4) can be demonstrated as:

28 $$\begin{aligned} \nu (\zeta )=\varpi _0 +\sum _{\sigma =1}^{\varsigma }\frac{\varpi _\sigma +\beta _\sigma \varphi ^{'}(\zeta )^\sigma }{\varphi (\zeta )^\sigma }. \end{aligned}$$

The solution to Eq. (4) can be found by calculating the constants $\varpi _0$, $\varpi _\sigma ,$ and $\beta _\sigma$ (where $\sigma =1,2,...,\varsigma$), the number $\varsigma$ is calculated by the homogeneous balance rule. The function $\varphi (\zeta )$ fulfills the subsequent evolution equation:

29 $$\begin{aligned} \varphi ^{'}(\zeta )^2=\varphi (\zeta )^2-\mu . \end{aligned}$$

By solving Eq. (29), one obtain the solution:

30 $$\begin{aligned} \varphi (\zeta )=a e^\zeta +\frac{\mu }{4a e^\zeta }, \end{aligned}$$

Where $\mu$ and *a* can take on any constant value.

Now, by plugging (28) along (29) into (4), the system of equations is established, the values of the constants can be obtained by solving it.

## Wave equation for QC-FNLSE

This section comprises mathematical analysis and convert the space-time QC-FNLSE into a nonlinear ODE. To solve Eq. (1), we utilize the fractional traveling wave transformation:

31 $$\begin{aligned} \psi (x,t)=e^{\iota \vartheta } \mu (\zeta ),~\zeta (x,t)=\frac{(x^\gamma +\varrho t^\gamma )}{\gamma },~\vartheta (x,t)=\frac{(-\tau x^\gamma +\omega t^\gamma )}{\gamma }. \end{aligned}$$

Inserting Eq. (31) into Eq. (1) and after separation, the imaginary part to serve is $\varrho =2\pi \tau$ and the real part to be:

32 $$\begin{aligned} \pi \mu {''}-(\omega +\pi \tau ^2)\mu -\eta \mu ^2+\lambda \mu ^3=0. \end{aligned}$$

In the transformation (31), the variable $\mu (\zeta )$ indicates the amplitude of the wave. The symbol $\varrho$ indicates the velocity of the wave, $\tau$ depicts the frequency of the soliton, and $\omega$ indicates the wave number.

## Formulation of solutions

### Application of the MSSM

Eq. (32) yields $\varsigma =1$ when $\mu ''$ and $\mu ^3$ are balanced. So from (5), we have following solutions

33 $$\begin{aligned} \mu (\zeta )=\Upsilon _{0}+\Upsilon _{1}\phi (\zeta ),~~~~\Upsilon _{1}\ne 0, \end{aligned}$$

Inserting the Eq. (6) and Eq. (33) into Eq. (32) yields a polynomial in $\phi ^r(\zeta )$. By grouping components with similar exponents and equating the resulting expression to zero, we establish a system of algebraic equations as;

34 $$\begin{aligned} & \lambda \Upsilon _0^3-\eta \Upsilon _0^2-\omega \Upsilon _0-\tau ^2 \Upsilon _0 \pi =0,\nonumber \\ & \varrho _1 \Upsilon _1 \pi +3 \lambda \Upsilon _0^2 \Upsilon _1-2 \eta \Upsilon _0 \Upsilon _1-\omega \Upsilon _1-\tau ^2 \Upsilon _1 \pi =0,\nonumber \\ & 3 \lambda \Upsilon _0 \Upsilon _1^2-\eta \Upsilon _1^2=0,\nonumber \\ & 2 \varrho _2 \Upsilon _1 \pi +\lambda \Upsilon _1^3=0. \end{aligned}$$

By performing straightforward manipulations, the unknown parameters can be determined by solving the above system of algebraic equations.

35 $$\begin{aligned} \Upsilon _0= \frac{\eta }{3 \lambda },~\omega = \frac{-9 \lambda \tau ^2 \pi -2 \eta ^2}{9 \lambda },~\varrho _2= -\frac{\lambda \Upsilon _1^2}{2 \pi },~\varrho _1= \frac{\eta ^2}{9 \lambda \pi }. \end{aligned}$$

By utilizing the solutions provided in Eq. (35) along with Eqs. (7)-(27) and (33), we may ascertain the novel soliton solutions of Eq. (1) in the following manner:

1. For $$\varrho _0=0,~\varrho _1>0,$$ and $$\varrho _2\ne 0$$.

The following are the bright and singular solitons, respectively:

36 $$\begin{aligned} \psi _1(x,t)=e^{\iota \vartheta } \left( \frac{\eta }{3 \lambda } \pm \Upsilon _1 \left( \frac{\sqrt{2}}{3} \sqrt{\frac{\eta ^2}{\lambda ^2 \Upsilon _1^2}}\right) \text {sech}\left( \left( \frac{1}{3} \sqrt{\frac{\eta ^2}{\lambda \pi }}\right) (\rho +\zeta )\right) \right) , \end{aligned}$$

**Fig. 1:**
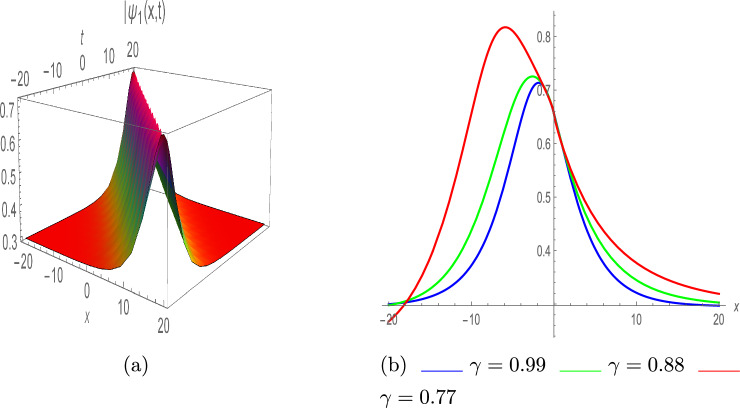
Bright soliton of $|\psi _1(x,t)|$, with $\eta =0.86,~ \lambda =-0.97,~ \tau =0.84,~ \Upsilon _1=-0.76,~ \pi =-0.94,~and~ \varrho =1$. (**a**) III-dimensional plot, (**b**) II-dimensional plot.

Figure 1 depicts the graphs of $\psi _1(x,t)$ with various parameter values; $\eta =0.86,~ \lambda =-0.97,~ \tau =0.84,~ \Upsilon _1=-0.76,~ \pi =-0.94,~and~ \varrho =1$. The graph in Fig. 1a displays the absolute value of the function $\psi _1(x,t)$, which has a bright soliton profile characterized by a high amplitude at its peak. Figure 1b depicts the 2D representation with various values of $\gamma$ such as 0.99, 0.88 and 0.77.

37 $$\begin{aligned} \psi _2(x,t)=e^{\iota \vartheta } \left( \frac{\eta }{3 \lambda } \pm \Upsilon _1 \left( \frac{\sqrt{2}}{3} \sqrt{-\frac{\eta ^2}{\lambda ^2 \Upsilon _1^2}}\right) \text {csch}\left( \left( \frac{1}{3} \sqrt{\frac{\eta ^2}{\lambda \pi }}\right) (\rho +\zeta )\right) \right) . \end{aligned}$$

**Fig. 2:**
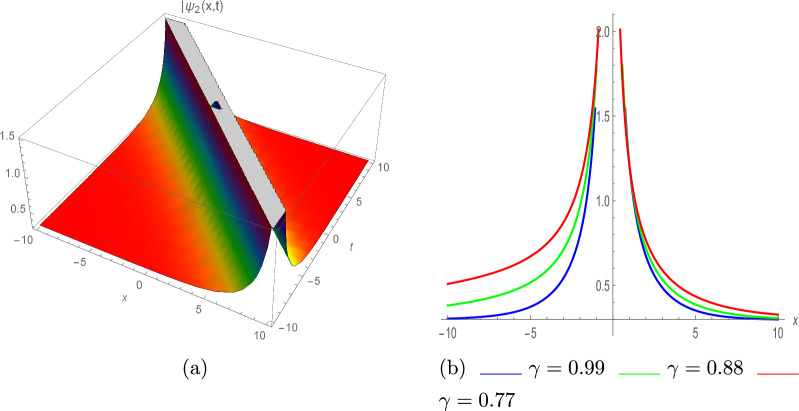
Singular soliton of $|\psi _2(x,t)|$, with $\eta =0.86,~\lambda =-0.97,~\tau =-0.84,~ \Upsilon _1=-0.76,~\pi =-0.94,$ and $\varrho =1$. (**a**) III-dimensional plot, (**b**) II-dimensional plot.

The graph in Fig. 2a displays the absolute value of $\psi _2(x,t)$ with distinct parameter values $\eta =0.86,~ \lambda =-0.97,~\tau =-0.84,~ \Upsilon _1=-0.76,~\pi =-0.94,$ and $\varrho =1$, highlighting the singular soliton profile characterized by localized behavior and singular properties. Figure 2b depicts the 2D illustration with various values of $\gamma$ such as 0.99, 0.88 and 0.77, portraying a visual representation of the singular soliton solutions. 2. For $\varrho _0=0,~\varrho _1>0,~\varrho _2=\pm 4\beta _1 \beta _2$, where $\beta _1,~\beta _2$ are constants.

The combo of bright-singular solitons is provided as:

38 $$\begin{aligned} \psi _3(x,t)= & e^{\iota \vartheta } \left( \frac{1}{3}\right. \nonumber \\ & \left. \left( \frac{\pm 4 \sqrt{\frac{\eta ^2}{\lambda \pi }}\beta _1 \Upsilon _1}{\left( 4 \beta _1^2+\frac{\lambda \Upsilon _1^2}{2 \pi }\right) \cosh \left( \left( \frac{1}{3} \sqrt{\frac{\eta ^2}{\lambda \pi }}\right) (\rho +\zeta )\right) \pm \left( 4 \beta _1^2-\frac{\lambda \Upsilon _1^2}{2 \pi }\right) \sinh \left( \left( \frac{1}{3} \sqrt{\frac{\eta ^2}{\lambda \pi }}\right) (\rho +\zeta )\right) }\right. \right. \nonumber \\ & \left. \left. +\frac{\eta }{\lambda }\right) \right) . \end{aligned}$$

3. For $$\varrho _0=\frac{\varrho _1^2}{4 \varrho _2},~\varrho _1<0,$$ and $$\varrho _2>0$$.

Below the dark and other singular solitons are given as:

39 $$\begin{aligned} \psi _4(x,t)=e^{\iota \vartheta } \left( \frac{\eta }{3 \lambda } \pm \Upsilon _1 \left( \frac{1}{3} \sqrt{\frac{\eta ^2}{\lambda ^2 \Upsilon _1^2}}\right) \tanh \left( \frac{\sqrt{-\frac{\eta ^2}{\lambda \pi }}}{3 \sqrt{2}}(\rho +\zeta )\right) \right) , \end{aligned}$$

**Fig. 3:**
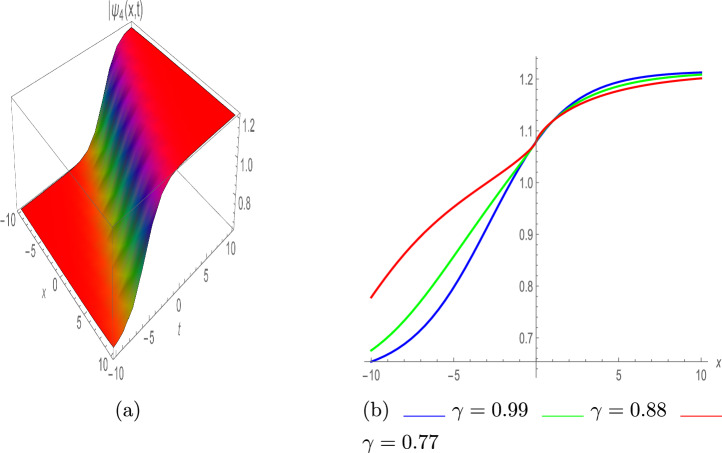
Kink soliton of $|\psi _4(x,t)|$, with $\eta =0.86,~ \lambda =-0.97,~ \tau =0.84,~ \Upsilon _0=-0.92,~ \omega _1=-0.76,~ \varrho =2$ and $\pi =0.94.$ (**a**) III-dimensional plot, (**b**) II-dimensional plot.

In Fig. 3a, the graph of $\psi _4(x,t)$ is presented in absolute value, illustrating the kink soliton profile with various parameter values $\eta =0.86,~ \lambda =-0.97,~ \tau =0.84,~ \Upsilon _0=-0.92,~ \Upsilon _1=-0.76,~ \varrho =2$ and $\pi =0.94.$. Figure 3b depicts the 2D illustration with various values of $\gamma$ such as 0.99, 0.88 and 0.77.

40 $$\begin{aligned} \psi _5(x,t)=e^{\iota \vartheta } \left( \frac{\eta }{3 \lambda } \pm \Upsilon _1 \left( \frac{1}{3} \sqrt{\frac{\eta ^2}{\lambda ^2 \Upsilon _1^2}}\right) \coth \left( \frac{\sqrt{-\frac{\eta ^2}{\lambda \pi }}}{3 \sqrt{2}}(\rho +\zeta )\right) \right) , \end{aligned}$$

The combined soliton solutions are as below:

41 $$\begin{aligned} \psi _6(x,t)= & e^{\iota \vartheta } \left( \frac{\eta }{3 \lambda }+\Upsilon _1 \left( \pm \left( \frac{1}{3} \sqrt{\frac{\eta ^2}{\lambda ^2 \Upsilon _1^2}}\right) \tanh \left( \left( \frac{\sqrt{2}}{3} \sqrt{-\frac{\eta ^2}{\lambda \pi }}\right) (\rho +\zeta )\right) \right. \right. \nonumber \\ & \left. \left. \pm i \text {sech}\left( \left( \frac{\sqrt{2}}{3} \sqrt{-\frac{\eta ^2}{\lambda \pi }}\right) (\rho +\zeta )\right) \right) \right) , \end{aligned}$$

42 $$\begin{aligned} \psi _7(x,t)= & e^{\iota \vartheta } \left( \frac{\eta }{3 \lambda } +\Upsilon _1 \tanh \left( \frac{\sqrt{-\frac{\eta ^2}{\lambda \pi }}}{6 \sqrt{2}}(\rho +\zeta )\right) \right. \nonumber \\ & \left. \left( \coth ^2\left( \frac{\sqrt{-\frac{\eta ^2}{\lambda \pi }}}{6 \sqrt{2}}(\rho +\zeta )\right) \pm \frac{1}{6} \sqrt{\frac{\eta ^2}{\lambda ^2 \Upsilon _1^2}}\right) \right) , \end{aligned}$$

**Fig. 4:**
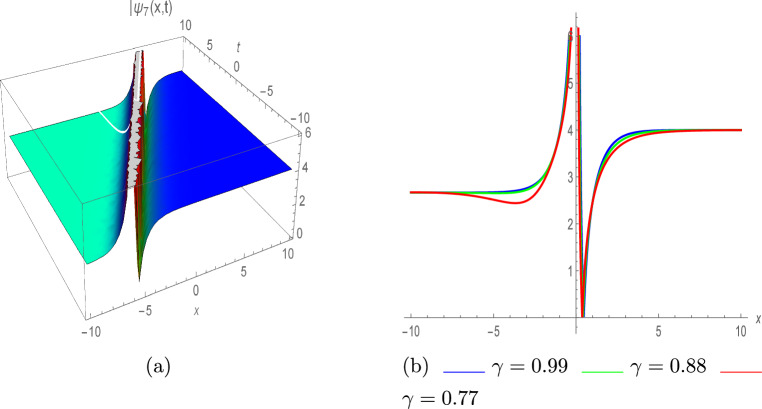
Mixed dark-singular soliton of $|\psi _7(x,t)|$, with $\eta =1,~ \lambda =-0.1,~ \tau =0.47,~ \Upsilon _1=-0.56,~ \pi =0.31,~ \varrho =-0.54, \Upsilon _0=-3.33,~\varrho _1=-3.58,~\varrho _2=0.05.$ (**a**) III-dimensional plot, (**b**) II-dimensional plot.

Figure 4a depicts the plot of absolute value $\psi _7(x,t)$ with various values of parameters; $\eta =1,~ \lambda =-0.1,~ \tau =0.47,~ \Upsilon _1=-0.56,~ \pi =0.31,~ \varrho =-0.54, \Upsilon _0=-3.33,~\varrho _1=-3.58,~\varrho _2=0.05.$ highlighting the combined dark-singular profile, where dark and singular solitons coexist. Figure 4b depicts the 2D illustration with various values of $\gamma$ such as 0.99, 0.88 and 0.77, showcasing how the system behaves dynamically.

43 $$\begin{aligned} \psi _8(x,t)= & e^{\iota \vartheta } \left( \frac{\eta }{3 \lambda }\right. \nonumber \\ & \pm \left. \frac{\Upsilon _1 \left( \pm \left( \frac{1}{3} \sqrt{\frac{\eta ^2}{\lambda ^2 \Upsilon _1^2}}\right) \right) \left( \sqrt{A_1^2+A_2^2}-A_1 \cosh \left( \left( \frac{\sqrt{2}}{3} \sqrt{-\frac{\eta ^2}{\lambda \pi }}\right) (\rho +\zeta )\right) \right) }{A_1 \sinh \left( \left( \frac{\sqrt{2}}{3} \sqrt{-\frac{\eta ^2}{\lambda \pi }}\right) (\rho +\zeta )\right) +A_2}\right) , \end{aligned}$$

44 $$\begin{aligned} \psi _9(x,t)= & e^{\iota \vartheta } \left( \frac{\eta }{3 \lambda }+\frac{\Upsilon _1 \left( \pm \left( \frac{1}{3} \sqrt{\frac{\eta ^2}{\lambda ^2 \Upsilon _1^2}}\right) \right) \cosh \left( \left( \frac{\sqrt{2}}{3} \sqrt{-\frac{\eta ^2}{\lambda \pi }}\right) (\rho +\zeta )\right) }{\sinh \left( \left( \frac{\sqrt{2}}{3} \sqrt{-\frac{\eta ^2}{\lambda \pi }}\right) (\rho +\zeta )\right) \pm i}\right) . \end{aligned}$$

4. For $$\varrho _0=0,~\varrho _1<0,$$ and $$\varrho _2\ne 0$$.

Following are the trigonometric function solutions:

45 $$\begin{aligned} \psi _{10}(x,t)= & e^{\iota \vartheta } \left( \frac{\eta }{3 \lambda }\pm \frac{1}{3} \Upsilon _1 \left( \sqrt{2} \sqrt{\frac{\eta ^2}{\lambda ^2 \Upsilon _1^2}}\right) \sec \left( \left( \frac{1}{3} \sqrt{-\frac{\eta ^2}{\lambda \pi }}\right) (\rho +\zeta )\right) \right) , \end{aligned}$$

**Fig. 5:**
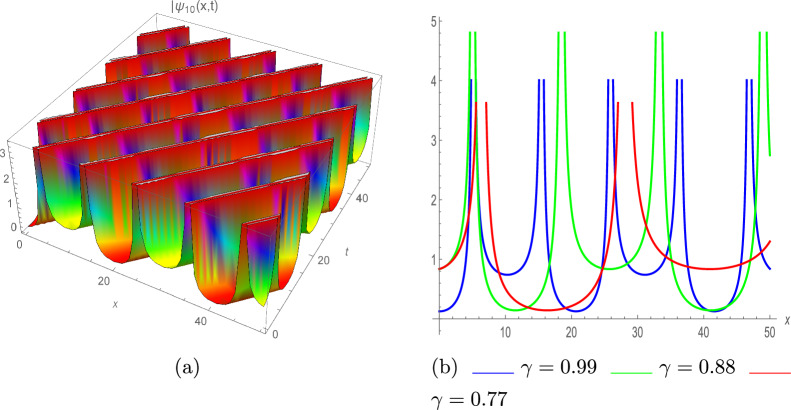
Periodic soliton of $|\psi _{10}(x,t)|$, with $\eta =0.81,~ \lambda =-0.88,~ \tau =0.84,~ \Upsilon _1=0.89,~ \pi =0.84,$ and $\varrho =-0.89.$ (**a**) III-dimensional plot, (**b**) II-dimensional plot.

Figure 5 demonstrates plots $\psi _{10}(x,t)$ with various values of parameters: $\eta =0.81,~ \lambda =-0.88,~ \tau =0.84,~ \Upsilon _1=0.89,~ \pi =0.84,$ and $\varrho =-0.89.$. In Fig. 5a, the graph of $\psi _{10}(x,t)$ is shown in absolute value, highlighting the periodic-singular soliton profile. Figure 5b depicts the 2D illustration with various values of $\gamma$ such as 0.99, 0.88 and 0.77, illustrating the periodic singular soliton solutions, demonstrating the interplay between periodicity and singularity.

46 $$\begin{aligned} \psi _{11}(x,t)= & e^{\iota \vartheta } \left( \frac{\eta }{3 \lambda }+ \Upsilon _1 \left( \pm \left( \frac{\sqrt{2}}{3} \sqrt{\frac{\eta ^2}{\lambda ^2 \Upsilon _1^2}}\right) \right) \csc \left( \left( \frac{1}{3} \sqrt{-\frac{\eta ^2}{\lambda \pi }}\right) (\rho +\zeta )\right) \right) . \end{aligned}$$

5. For $$\varrho _0=\frac{\varrho _1^2}{4 \varrho _2},\varrho _1>0,\varrho _2>0,$$ and $$A_1^2-A_2^2>0$$.

47 $$\begin{aligned} \psi _{12}(x,t)= & e^{\iota \vartheta } \left( \frac{\eta }{3 \lambda }\pm \frac{1}{3} \Upsilon _1 \sqrt{-\frac{\eta ^2}{\lambda ^2 \Upsilon _1^2}} \tan \left( \frac{\sqrt{\frac{\eta ^2}{\lambda \pi }}}{3 \sqrt{2}}(\rho +\zeta )\right) \right) , \end{aligned}$$

48 $$\begin{aligned} \psi _{13}(x,t)= & e^{\iota \vartheta } \left( \frac{\eta }{3 \lambda }+\Upsilon _1 \left( \pm \left( \frac{1}{3} \sqrt{-\frac{\eta ^2}{\lambda ^2 \Upsilon _1^2}}\right) \right) \cot \left( \frac{\sqrt{\frac{\eta ^2}{\lambda \pi }}}{3 \sqrt{2}}(\rho +\zeta )\right) \right) , \end{aligned}$$

Below are the mixed trigonometric function solutions:

49 $$\begin{aligned} \psi _{14}(x,t)= & e^{\iota \vartheta } \left( \frac{\eta }{3 \lambda }+\Upsilon _1 \left( \pm \left( \frac{1}{3} \sqrt{-\frac{\eta ^2}{\lambda ^2 \Upsilon _1^2}}\right) \tan \left( \left( \frac{\sqrt{2}}{3} \sqrt{\frac{\eta ^2}{\lambda \pi }}\right) (\rho +\zeta )\right) \right. \right. \nonumber \\ & \left. \left. \pm \sec \left( \left( \frac{\sqrt{2}}{3} \sqrt{\frac{\eta ^2}{\lambda \pi }}\right) (\rho +\zeta )\right) \right) \right) , \end{aligned}$$

50 $$\begin{aligned} \psi _{15}(x,t)= & e^{\iota \vartheta } \left( \frac{\eta }{3 \lambda }+ \Upsilon _1 \tan \left( \frac{\sqrt{\frac{\eta ^2}{\lambda \pi }}}{6 \sqrt{2}}(\rho +\zeta )\right) \left( -\cot ^2\left( \frac{\sqrt{\frac{\eta ^2}{\lambda \pi }}}{6 \sqrt{2}}(\rho +\zeta )\right) \right. \right. \nonumber \\ & \left. \left. \pm \frac{1}{6} \sqrt{-\frac{\eta ^2}{\lambda ^2 \Upsilon _1^2}}\right) \right) , \end{aligned}$$

51 $$\begin{aligned} \psi _{16}(x,t)= & e^{\iota \vartheta } \left( \frac{\eta }{3 \lambda }+\Upsilon _1 \pm \left( \frac{1}{3} \sqrt{-\frac{\eta ^2}{\lambda ^2 \Upsilon _1^2}}\right) \right. \nonumber \\ & \left. \left( \pm \frac{\sqrt{A_1^2-A_2^2}-A_1 \cos \left( \left( \frac{\sqrt{2}}{3} \sqrt{\frac{\eta ^2}{\lambda \pi }}\right) (\rho +\zeta )\right) }{A_1 \sin \left( \left( \frac{\sqrt{2}}{3} \sqrt{\frac{\eta ^2}{\lambda \pi }}\right) (\rho +\zeta )\right) +A_2}\right) \right) , \end{aligned}$$

52 $$\begin{aligned} \psi _{17}(x,t)= & e^{\iota \vartheta } \left( \frac{\eta }{3 \lambda }+\frac{\Upsilon _1 \left( \pm \left( \frac{1}{3} \sqrt{-\frac{\eta ^2}{\lambda ^2 \Upsilon _1^2}}\right) \right) \cos \left( \left( \frac{\sqrt{2}}{3} \sqrt{\frac{\eta ^2}{\lambda \pi }}\right) (\rho +\zeta )\right) }{\sin \left( \left( \frac{\sqrt{2}}{3} \sqrt{\frac{\eta ^2}{\lambda \pi }}\right) (\rho +\zeta )\right) \pm 1}\right) . \end{aligned}$$

6. For $$\varrho _0=0,$$ and $$\varrho _1>0$$.

The exponential solutions are obtain as:

53 $$\begin{aligned} \psi _{18}(x,t)= & e^{\iota \vartheta } \left( \frac{9 \eta \pi ^2~ e^{\pm 2 \left( \frac{1}{3} \sqrt{\frac{\eta ^2}{\lambda \pi }}\right) (\rho +\zeta )}+12 \eta ^2 \Upsilon _1 \pi ~ e^{\pm \left( \frac{1}{3} \sqrt{\frac{\eta ^2}{\lambda \pi }}\right) (\rho +\zeta )}+2 \eta ^3 \Upsilon _1^2}{27 \lambda \pi ^2 ~e^{\pm 2 \left( \frac{1}{3} \sqrt{\frac{\eta ^2}{\lambda \pi }}\right) (\rho +\zeta )}+6 \eta ^2 \lambda \Upsilon _1^2}\right) , \end{aligned}$$

54 $$\begin{aligned} \psi _{19}(x,t)= & e^{\iota \vartheta } \left( \frac{2 \eta ^3 \Upsilon _1^2 ~e^{\pm 2 \left( \frac{1}{3} \sqrt{\frac{\eta ^2}{\lambda \pi }}\right) (\rho +\zeta )}\pm 12 \eta ^2 \Upsilon _1 \pi ~e^{\pm \left( \frac{1}{3} \sqrt{\frac{\eta ^2}{\lambda \pi }}\right) (\rho +\zeta )}+9 \eta \pi ^2}{6 \eta ^2 \lambda \Upsilon _1^2 ~e^{\pm 2 \left( \frac{1}{3} \sqrt{\frac{\eta ^2}{\lambda \pi }}\right) (\rho +\zeta )}+27 \lambda \pi ^2}\right) . \end{aligned}$$

The rational solutions are obtained as:

7. For $$\varrho _0=\varrho _1=0,$$ and $$\varrho _2>0$$.

55 $$\begin{aligned} \psi _{20}(x,t)= & e^{\iota \vartheta }\left( \frac{\eta }{3 \lambda } +\frac{\Upsilon _1 \pm 1}{\frac{\sqrt{-\frac{\lambda \Upsilon _1^2}{\pi }}}{\sqrt{2}}(\rho +\zeta )}\right) . \end{aligned}$$

8. For $$\varrho _0=\varrho _1=0,$$ and $$\varrho _2<0$$.

56 $$\begin{aligned} \psi _{21}(x,t)= & e^{\iota \vartheta } \left( \frac{\eta }{3 \lambda }+\frac{\Upsilon _1 \pm i}{\frac{\sqrt{\frac{\lambda \Upsilon _1^2}{\pi }}}{\sqrt{2}}(\rho +\zeta )}\right) . \end{aligned}$$

### Application of $(\frac{1}{\varphi (\zeta )},\frac{\varphi ^{'}(\zeta }{\varphi (\zeta )})$ Method

After applying the homogeneous balance rule on Eq. (32), $\varsigma =1$ is obtained. Based on (28), we thus obtain

57 $$\begin{aligned} \mu (\zeta )=\varpi _0+\frac{\varpi _1+\beta _1 \varphi ^{'}(\zeta )}{\varphi (\zeta )}. \end{aligned}$$

Eq. (57) along with Eq. (29) is plugged into Eq. (32) to provide a set of polynomial equations as;

58 $$\begin{aligned} & 3 \beta _1^2 \lambda \varpi _0-\beta _1^2 \eta +\lambda \varpi _0^3-\eta \varpi _0^2-\omega \varpi _0-\tau ^2 \varpi _0 \pi =0,\nonumber \\ & -3 \beta _1^2 \lambda \sigma \varpi _1+\lambda \varpi _1^3-2 \sigma \varpi _1 \pi =0,\nonumber \\ & -3 \beta _1^2 \lambda \sigma \varpi _0+\beta _1^2 \eta \sigma -\eta \varpi _1^2+3 \lambda \varpi _0 \varpi _1^2=0,\nonumber \\ & 3 \beta _1^2 \lambda \varpi _1+3 \lambda \varpi _0^2 \varpi _1-2 \eta \varpi _0 \varpi _1-\omega \varpi _1-\tau ^2 \varpi _1 \pi +\varpi _1 \pi =0,\nonumber \\ & -2 \beta _1 \sigma \pi -\beta _1^3 \lambda \sigma +3 \beta _1 \lambda \varpi _1^2=0,\nonumber \\ & 6 \beta _1 \lambda \varpi _0 \varpi _1-2 \beta _1 \eta \varpi _1=0,\nonumber \\ & -\beta _1 \tau ^2 \pi +3 \beta _1 \lambda \varpi _0^2-2 \beta _1 \eta \varpi _0+\beta _1^3 \lambda -\beta _1 \omega =0, \end{aligned}$$

The following set of solutions are attained by solving the above system.

**Set 1**

59 $$\begin{aligned} \left\{ \varpi _0= \frac{\sqrt{\pi }}{\sqrt{\lambda }},~\varpi _1= \pm \frac{\sqrt{2} \sqrt{\sigma } \sqrt{\pi }}{\sqrt{\lambda }},~\beta _1= 0,~\varpi = -\tau ^2 \pi -2 \pi ,~\eta = 3 \sqrt{\lambda } \sqrt{\pi } \right\} . \end{aligned}$$

By plugging Eq. (59) with Eq. (30) into Eq. (57), we acquire the following solutions;

60 $$\begin{aligned} \varphi (x,t)=e^{\iota \vartheta } \left( \frac{\sqrt{\pi }}{\sqrt{\lambda }}\pm \frac{\sqrt{2} \sqrt{\sigma } \sqrt{\pi }}{\sqrt{\lambda } \left( \sigma e^{-\zeta }+4a^2 e^\zeta \right) }\right) . \end{aligned}$$

By plugging $\sigma =\pm 4a^2$, above Eq. yields

61 $$\begin{aligned} \varphi _{1,1}(x,t)=e^{\iota \vartheta } \left( \frac{\sqrt{\pi }}{\sqrt{\lambda }}\pm \frac{\sqrt{2} \sqrt{a^2} \sqrt{\pi } ~\text {sech}(\zeta )}{a \sqrt{\lambda }}\right) , \end{aligned}$$

and

62 $$\begin{aligned} \varphi _{1,2}(x,t)=e^{\iota \vartheta } \left( \frac{\sqrt{\pi }}{\sqrt{\lambda }}\pm \frac{\sqrt{2} \sqrt{-a^2} \sqrt{\pi } ~\text {csch}(\zeta )}{a \sqrt{\lambda }}\right) . \end{aligned}$$

**Fig. 6:**
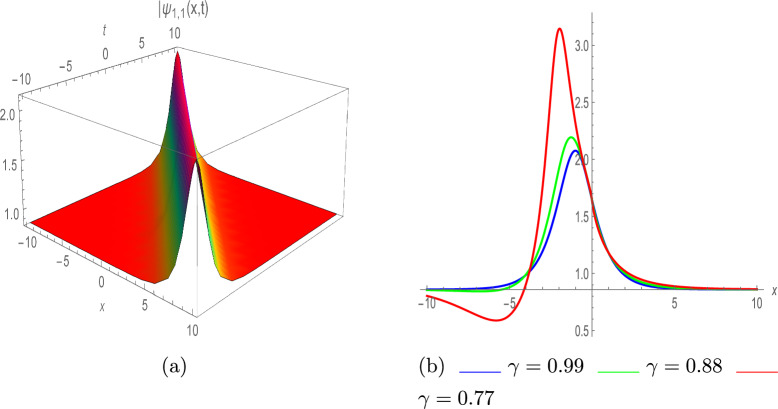
Bright soliton of $|\psi _{1,1}(x,t)|$, with $\pi =0.34,~a=-0.53,~ \lambda =0.46,~ \varrho =1.$ (**a**) III-dimensional plot, (**b**) II-dimensional plot.

In Fig. 6, plot of absolute value of $\psi _{1,1}(x,t)$ is drawn with various parameter values; $a=-0.53,~ \lambda =0.46,~ \pi =0.34,~ and ~\varrho =1$, demonstrating the bright soliton profile. Figure 6b depicts the 2D illustration with various values of $\gamma$ such as 0.99, 0.88 and 0.77.

**Set 2**

63 $$\begin{aligned} \left\{ \varpi _0= -\frac{6 \pi }{\eta },~\varpi _1= 0,~\beta _1= \pm \frac{6 \pi }{\eta },~\varpi = 4 \pi -\tau ^2 \pi ,~\lambda = -\frac{\eta ^2}{18 \pi }\right\} . \end{aligned}$$

By plugging Eq. (63) with Eq. (30) into Eq. (57), we get the below solutions;

64 $$\begin{aligned} \varphi (x,t)=e^{\iota \vartheta } \left( -\frac{6 \pi }{\eta }\pm \frac{6 \pi \left( 4a^2 e^\zeta -\sigma e^{-\zeta }\right) }{\eta \left( \sigma e^{-\zeta }+4a^2 e^\zeta \right) }\right) . \end{aligned}$$

By plugging $\sigma =\pm 4a^2$, above Eq. yields

65 $$\begin{aligned} \varphi _{2,1}(x,t)=e^{\iota \vartheta } \left( -\frac{6 \pi }{\eta }\pm \frac{6 \pi \tanh (\zeta )}{\eta }\right) , \end{aligned}$$

and

66 $$\begin{aligned} \varphi _{2,2}(x,t)=e^{\iota \vartheta } \left( -\frac{6 \pi }{\eta }\pm \frac{6 \pi \coth (\zeta )}{\eta }\right) . \end{aligned}$$

**Fig. 7:**
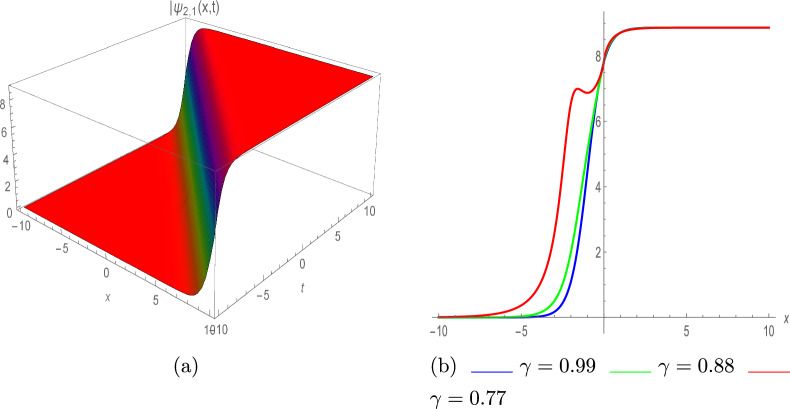
Kink soliton of $|\psi _{2,1}(x,t)|$, with $\eta =0.46,~ \pi =0.34,~ and~ \varrho =1.$ (**a**) III-dimensional plot, (**b**) II-dimensional plot.

Figure 7a shows the graph of absolute value of $\psi _{2,1}(x,t)$ showcasing the kink soliton profile with various parameter values; $\eta =0.46,~ \pi =0.34,~ and~ \varrho =1$. Fig. 7b depicts the 2D illustration with various values of $\gamma$ such as 0.99, 0.88 and 0.77, showcasing the dynamic behavior of system.

## The numerical solution employing the differential transform method (DTM)

The DTM is a semi-analytical and numerical technique used to solve a wide range of differential equations, typically producing solutions in series form. Originally proposed by Zhou^57^ for addressing linear and nonlinear initial value problems in electric circuit analysis, DTM has since become a valuable tool for solving ordinary differential equations due to its fast convergence rate and small calculation error. A key advantage of DTM over integral transformation methods is its capability to effectively handle nonlinear differential equations, including both initial value and boundary value problems. The following describes the differential transform of the $j^{th}$ derivative of the function *h*(*x*):

67 $$\begin{aligned} H(j)=\frac{1}{j!}\left[ \frac{d^j h(x)}{dx^j}\right] _{x=0}, \end{aligned}$$

where *h*(*x*) is the original function, and *H*(*j*) is the transformed function. The differential inverse transform of *H*(*j*) is defined as

68 $$\begin{aligned} h(x)=\sum _{j=0}^\infty H(j) x^j \approx h_N(x)=\sum _{j=0}^N H(j) x^j. \end{aligned}$$

By substituting eq. (67) in (68) we get

69 $$\begin{aligned} h(x)=\sum _{j=0}^\infty \frac{x^j}{j!}\frac{d^j h(x)}{dx^j}\big |_{x=0}. \end{aligned}$$

In the preceding formulation, we examine the scenario when $x = 0$; however, this is applicable to any fixed real number $x = x_0$. The key results obtained from eqs. (67) and (68) can be found in^58–60^.

### Applications and numerical results

**DTM solution of achieved solution by using the MSSM**

This section presents the numerical methodology for deriving the wave solutions of Eq. (32), based on the obtained results of the MSSM with the corresponding parameter values.

For simulating the bright soliton described in Eq. (36), we assume the following fixed real values for the involved parameters: $\eta =3,~ \lambda =-1,~ \tau =1,~ \omega _1=1,~ \pi =-1,~ \omega =3.$

Eq. (32) reduces to

70 $$\begin{aligned} - \mu {''}-2\mu -3 \mu ^2- \mu ^3=0. \end{aligned}$$

From Eq. (36), the solution of Eq. (70) with the initial conditions is:

71 $$\begin{aligned} \mu (\zeta )= & \sqrt{2}~ \text {sech}(\zeta +1)-1, \end{aligned}$$

where $$\zeta =~\frac{(x^\gamma +\varrho t^\gamma )}{\gamma }$$, and

72 $$\begin{aligned} & \mu (0)=\sqrt{2}~~ \text {sech}(1)-1,\\ & \nonumber \mu '(0)=-\sqrt{2} \tanh (1) \text {sech}(1). \end{aligned}$$

Applying DTM on (70), we obtain

73 $$\begin{aligned} -(j+1)(j+2)H(j+2)-2H(j)-3F(j)-G(j)=0, \end{aligned}$$

where the transformation function *H*(*j*) represents the original function $\mu (\zeta )$, and the transformation for the nonlinear terms $\mu ^{2}$ and $\mu ^{3}$ is denoted as *F*(*j*) and *G*(*j*) respectively, defined as

74 $$\begin{aligned} F(j)={\left\{ \begin{array}{ll} H^m(0), & \text {if j=0},\\ \frac{1}{H(0)} \sum _{r=0}^j \frac{(m+1)r-j}{j} H(r) F(j-r), & \text {if }\,\,j\ge 1, \end{array}\right. } \end{aligned}$$

where *m* denotes the power of nonlinear term. From (74) we get

75 $$\begin{aligned} H(j+2)= & \frac{-2H(j)-3F(j)-G(j)}{(j+1)(j+2)};~H(0)=\mu (0)=\sqrt{2}~~ \text {sech}(1)-1,\\ & \nonumber H(1)=\mu '(0)=-\sqrt{2} \tanh (1) \text {sech}(1). \end{aligned}$$

Plugging $j=0$, in Eq. (75), we attain

$$\begin{aligned} H(2)=\frac{-2H(0)-3F(0)-G(0)}{(1)(2)}=0.0733425. \end{aligned}$$

In the similar way, the coefficients *H*(*j*) can be achieved as

$$\begin{aligned} H(3)=0.176807,~H(4)=-0.120915. \end{aligned}$$

Then, with initial conditions (71), the DTM solution of Eq. (70) is written as

76 $$\begin{aligned} \mu (\zeta )=\sum _{j=0}^\infty H(j)\zeta ^j\Rightarrow \mu _N(\zeta )\cong \sum _{j=0}^N H(j)\zeta ^j=\sum _{j=0}^{10} H(j)\zeta ^j. \end{aligned}$$

Hence

77 $$\begin{aligned} \mu _{DTM}(\zeta )= & -0.0835129 -0.697991 \zeta +0.0733425 \zeta ^2+0.176807 \zeta ^3-0.120915 \zeta ^4 \\ & \nonumber +0.017642 \zeta ^5 +0.0246802 \zeta ^6 -0.0192707 \zeta ^7 +0.00399391 \zeta ^8 +0.00325196 \zeta ^9 \\ & \nonumber -0.00302725 \zeta ^{10}. \end{aligned}$$

This closely resembles the Maclaurin Taylor series expansion of the exact solution

78 $$\begin{aligned} \mu _{Exact}(\zeta )= & -0.0835129 -0.697991 \zeta +0.0733425 \zeta ^2 +0.176807 \zeta ^3 -0.120915 \zeta ^4 \\ & \nonumber +0.017642 \zeta ^5 +0.0246802 \zeta ^6 -0.0192707 \zeta ^7 +0.00399391 \zeta ^8 \\ & \nonumber +0.00325196 \zeta ^9 -0.00302725 \zeta ^{10}. \end{aligned}$$

Now using the DTM, the absolute error of the exact solution and the numerical solution is

79 $$\begin{aligned} \text {Error}= |\text {Exact solution - DTM solution}|= \begin{bmatrix} 0\\ 0\\ 2.77556 \times 10^{-17}\\ 0\\ 0\\ 0\\ 0\\ 0\\ 1.11022 \times 10^{-16}\\ 3.33067 \times 10^{-16}\\ 1.11022 \times 10^{-15} \end{bmatrix}. \end{aligned}$$

**Fig. 8:**
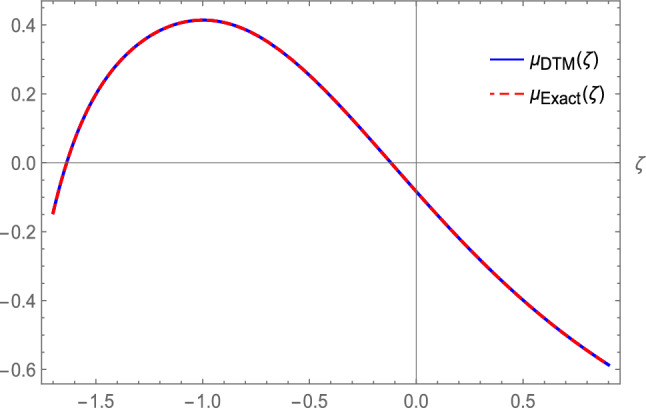
Comparison between the exact and DTM solution.

**DTM solution of achieved solution by using the** $(\frac{1}{\varphi (\zeta )},\frac{\varphi ^{'}(\zeta )}{\varphi (\zeta )})$ **method**

Here, we derived DTM solution of Eq. (65) with the following fixed parametric values: $\eta =6,~ \tau =1,~ \pi =1,~\omega =3,~\lambda =-2$.

Eq. (32) reduces to

80 $$\begin{aligned} \mu {''}-4\mu -6 \mu ^2-2 \mu ^3=0. \end{aligned}$$

From Eq. (65), the solution of Eq. (80) with the initial conditions is:

81 $$\begin{aligned} \mu (\zeta )= & -\tanh (\zeta +1)-1, \end{aligned}$$

where $$\zeta =~\frac{(x^\gamma +\varrho t^\gamma )}{\gamma }$$, and

82 $$\begin{aligned} & \mu (0)=-\tanh (1)-1, \\ & \nonumber \mu '(0)=-\text {sech}^2(1). \end{aligned}$$

Using initial conditions (81) and (74), the DTM solution of (80) is written as

83 $$\begin{aligned} \mu (\zeta )=\sum _{j=0}^\infty H(j)\zeta ^j\Rightarrow \mu _N(\zeta )\cong \sum _{j=0}^N H(j)\zeta ^j=\sum _{j=0}^{10} H(j)\zeta ^j. \end{aligned}$$

Hence

84 $$\begin{aligned} \mu _{DTM}(\zeta )= & -1.76159 -0.419974 \zeta +0.31985 \zeta ^2 -0.103604 \zeta ^3 -0.0277121 \zeta ^4 \\ & \nonumber +0.0463074 \zeta ^5 -0.0189223 \zeta ^6 -0.00243817 \zeta ^7 +0.00687158 \zeta ^8 \\ & \nonumber -0.00326119 \zeta ^9 -0.000100974 \zeta ^{10}. \end{aligned}$$

This closely resembles the Maclaurin Taylor series expansion of the exact solution

85 $$\begin{aligned} \mu _{Exact}(\zeta )= & -1.76159-0.419974 \zeta +0.31985 \zeta ^2 -0.103604 \zeta ^3 -0.0277121 \zeta ^4 \\ & \nonumber +0.0463074 \zeta ^5 -0.0189223 \zeta ^6 -0.00243817 \zeta ^7 +0.00687158 \zeta ^8 \\ & \nonumber -0.00326119 \zeta ^9 -0.000100974 \zeta ^{10}. \end{aligned}$$

Now using the DTM, the absolute error of the exact solution and the numerical solution is

86 $$\begin{aligned} \text {Error}= |\text {Exact solution - DTM solution}|= \begin{bmatrix} 4.88498 \times 10^{-15}\\ 8.88178 \times 10^{-16}\\ 3.77476 \times 10^{-15}\\ 8.88178 \times 10^{-16}\\ 3.55271 \times 10^{-15}\\ 3.10862 \times 10^{-15}\\ 3.9968 \times 10^{-15}\\ 1.55431 \times 10^{-15}\\ 1.11022 \times 10^{-15}\\ 3.55271 \times 10^{-15}\\ 3.9968 \times 10^{-15} \end{bmatrix}. \end{aligned}$$

**Fig. 9:**
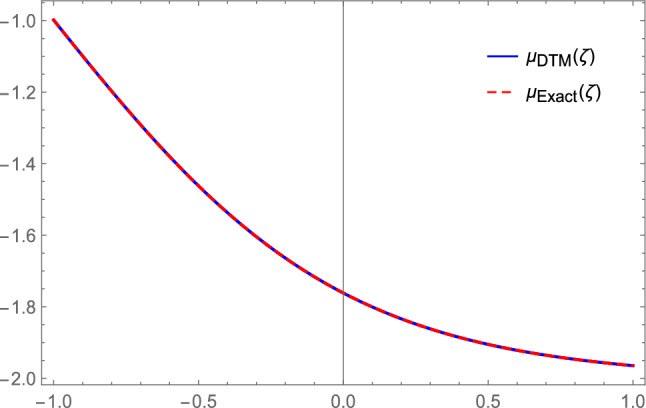
Comparison between the exact and DTM solution.

Figure 8, 9 demonstrates a numerical comparison of how comparable and accurate individual wave solutions derived analytically using the provided approaches are to DTM numerical solutions. A low inaccuracy and consistent agreement between analytical and numerical solutions demonstrate the proposed methods accuracy in modeling model dynamics.

**Convergence Analysis of DTM**

To evaluate the numerical reliability of the differential transform method, a rigorous convergence analysis is performed utilizing the maximum absolute error $E_N=max_{\zeta \in I} |\mu _N(\zeta )-\mu _{Exact}(\zeta )|$ on the interval $I=[-0.5,0.5]$ (the region where the soliton is localized in our plots). This error measures the point-wise deviation between the exact solution and the truncated DTM series.

For our problem, the computed maximum absolute errors are:

$$\begin{aligned} E_{max}=1.12 \times 10^{-15}~~~~~~~\text {for Eq. (79)},\\ E_{max}=4.96 \times 10^{-15}~~~~~~~\text {for Eq. (86)}. \end{aligned}$$

The values, approaching machine precision, indicate that the DTM approximation converges quickly to the exact solution and demonstrates high numerical accuracy. This illustrates the method’s reliability and robust convergence characteristics for the equations being analyzed.

## Results and discussion

Through the use of adequate transformation techniques, we have effectively obtained wave solutions for optical solitons and other solutions to the space and time fractional QC-FNLSE. The results that are provided could be useful for a method that is easy to understand when dealing with complicated nonlinear disciplines. The key significance of this study resides in its novel methodology for addressing the QC-FNLSE. By employing the proposed techniques, the research presents novel soliton solutions that enhance our comprehension of nonlinear wave phenomena. These findings are crucial for applications in nonlinear optics, ferromagnetic materials, plasma physics, and other emerging sciences. This section highlights the uniqueness of the current study by conducting a thorough comparison of the evaluated results with outcomes calculated in prior research. We show a comparison between our solutions and that obtained by Aslan *et*. *al*^61^ and Attia *et*. *al*^62^ as follows: Aslan *et*. *al* applied the Jacobi elliptic functions to the quadratic–cubic NLS equation when $\delta =1$. They obtained bright and dark optical soliton solutions. While Attia *et*. *al* applied two techniques and obtained only dark, singular and periodic soliton solutions. We obtain many different forms of solutions that are completely different from that obtained in Ref.^61,62^ that makes our solutions novel and considerable for publication. Moreover, we have examined the numerical solutions of this model. The DTM is applied to this model under specific boundary conditions obtained by using the resulting solutions of the analytically used methods. Using the DTM results, we evaluate the convergence and error behavior of the obtained solutions. The resulting comparison confirms the accuracy and consistency of the derived closed-form expressions. The solutions, such as bright, kink, periodic, singular, combined dark-singular and combined dark-bright are shown in II- and III-dimensional space to showcase their physical and material phenomena, emphasizing the solitons of different shapes. The parameter values employed in the figures are chosen based on physically pertinent regimes of optical fiber propagation and the mathematical limitations of the analytical techniques. The effective dispersion and quadratic–cubic nonlinearities of the QC–NLSE are represented by the coefficients $\pi ,~\eta$ and $\lambda$. The selected values fall within the usual ranges documented for practical fiber configurations. The solution parameters $\Upsilon _0$ and $\Upsilon _1$ are derived from the admissible sets that comply with the algebraic restrictions established by our proposed methods, guaranteeing that the resulting waveforms are real, bounded, and physically significant. The fractional order $\gamma$ is adjusted within the range (0,1] to demonstrate the impact of memory effects in fractional optical media. Consequently, all parameter combinations employed in the graphical representations align with physically interpretable and mathematically compatible propagation regimes.

**Physical significance of the obtained soliton solutions**

The analytical solutions presented in this study relate to various established pulse structures in nonlinear optics, each representing a distinct propagation mechanism in quadratic–cubic media. The bright soliton solutions indicate self-localized pulses that emerge when the effective dispersion and nonlinear coefficients achieve a focusing balance. Such solutions simulate steady, high-intensity optical pulses in fibers, where nonlinear self-phase modulation mitigates pulse broadening. Kink-type solutions, on the other hand, describe intensity dips that are embedded in a continuous-wave background. This is a phenomenon that happens in optical fibers associated with defocusing regimes. These structures encapsulate phase-shifted regions where the background field persists as nonzero, aligning with experimental findings in defocusing Kerr-type media.

The periodic wave solutions relate to nonlinear optical lattices or periodic pulse trains, emerging when the system allows for spatially or temporally recurring patterns. These solutions have relevance in optical communication systems where periodic modulation, wave mixing, or optical lattices influence pulse evolution.

The singular and dark–singular solutions mathematically characterize waveforms that display diverging amplitude at discrete places. Although these singularities do not directly relate to real optical pulses, they are crucial for comprehending the asymptotic behavior of the solution space, recognizing potential blow-up regimes, and delineating the limits of model validity. These solutions also function as benchmarks for identifying instabilities or potential structural changes as system parameters fluctuate.

Lastly, dark-bright mixed solutions are composite structures in which a localized bright component propagates concurrently with a dark background distortion. Such mixed modes are recognized in multi-component nonlinear optical systems and characterize the energy exchange among various wave channels, frequently observed in birefringent fibers, coupled-mode systems, and media exhibiting competing nonlinearities. The fractional order $\gamma$ of all these wave structures shows how memory and nonlocal effects work in the medium. For example, decreasing the order usually makes the pulse wider, changes its amplitude, and changes its speed of propagation, offering an extra level of control in fractional optical models.

## Conclusion

In this paper, We presented new closed-form optical soliton families of the fractional quadratic–cubic NLSE in the conformable derivative framework by applying the modified Sardar sub-equation method and the $(\frac{1}{\varphi (\zeta )},\frac{\varphi ^{'}(\zeta )}{\varphi (\zeta )})$ method. A large number of optical soliton solutions in various forms, including rational, trigonometric, and hyperbolic functions, are accumulated in this work. The diversity of optical soliton solutions exemplifies the richness and complexity of the optical phenomena addressed by this mathematical framework, which includes mixed dark-bright, singular, dark, bright, dark-singular and periodic singular optical solitons. The obtained soliton solutions possess various distinct physical interpretations. Furthermore, the 2D graphs generated using Mathematica illustrate the behaviors of the solutions for various fractional orders that captures nonlocal effects and modifies pulse width and amplitude systematically. Numerical validation using the differential transform method, supported by convergence and error analysis, confirmed the accuracy of the obtained solutions. These results broaden the soliton landscape of the fractional QC–NLSE and provide a foundation for future work on stability, experimental parameter mapping, and extensions to coupled or higher-dimensional fractional systems.

## Data Availability

No datasets were generated or analysed during the current study.

## References

[1] Miller, K. S. & Ross, B. *An Introduction to the Fractional Calculus and Fractional Differential Equations* (Wiley, New York, 1993).

[2] Xu, Y.-J., Bilal, M., Al-Mdallal, Q., Khan, M. A. & Muhammad, T. Gyrotactic micro-organism flow of maxwell nanofluid between two parallel plates. *Sci. Rep.* **11**(1), 15142 (2021). 34312440 10.1038/s41598-021-94543-4 PMC8313715

[3] Waqas, H., Farooq, U., Alqarni, M., Muhammad, T. & Khan, M. A. Bioconvection transport of magnetized micropolar nanofluid by a Riga plate with non-uniform heat sink/source. *Waves Random Complex Media* 1–20 (2021).

[4] Bhatti, M. M. & Lu, D. Q. Analytical study of the head-on collision process between hydroelastic solitary waves in the presence of a uniform current. *Symmetry* **11**(3), 333 (2019).

[5] Raza, N., Rafiq, M. H., Bekir, A. & Rezazadeh, H. Optical solitons related to (2+ 1)-dimensional Kundu–Mukherjee–Naskar model using an innovative integration architecture. *J. Nonlinear Opt. Phys. Mater.* **31**(03), 2250014 (2022).

[6] Vlase, S., Marin, M., Öchsner, A. & Scutaru, M. Motion equation for a flexible one-dimensional element used in the dynamical analysis of a multibody system. *Continuum Mech. Thermodyn.* **31**, 715–724 (2019).

[7] Bhatti, M. M. & Lu, D. Q. Hydroelastic solitary wave during the head-on collision process in a stratified fluid. *Proc. Inst. Mech. Eng. C J. Mech. Eng. Sci.* **233**(17), 6135–6148 (2019).

[8] Khater, M. M. Lax representation and bi-Hamiltonian structure of nonlinear GIAO model. *Mod. Phys. Lett. B* **36**(07), 2150614 (2022).

[9] Raza, N., Murtaza, I. G., Sial, S. & Younis, M. On solitons: The biomolecular nonlinear transmission line models with constant and time variable coefficients. *Waves Random Complex Media* **28**(3), 553–569 (2018).

[10] Khater, M. M., Muhammad, S., Al-Ghamdi, A. & Higazy, M. Abundant wave structures of the fractional Benjamin–Ono equation through two computational techniques. *J. Ocean Eng. Sci. *(2022).

[11] Alotaibi, M. F., Omri, M., Khalil, E., Abdel-Khalek, S., Bouslimi, J., & Khater, M. M. Abundant solitary and semi-analytical wave solutions of nonlinear shallow water wave regime model. *J. Ocean Eng. Sci.* (2022).

[12] Raza, N. & Javid, A. Dynamics of optical solitons with Radhakrishnan–Kundu–Lakshmanan model via two reliable integration schemes. *Optik* **178**, 557–566 (2019).

[13] Khater, M. M., Mohamed, M. S. & Attia, R. A. On semi analytical and numerical simulations for a mathematical biological model; The time-fractional nonlinear Kolmogorov–Petrovskii–Piskunov (KPP) equation. *Chaos Solitons Fractals* **144**, 110676 (2021).

[14] Park, C., Khater, M. M., Attia, R. A., Alharbi, W. & Alodhaibi, S. S. An explicit plethora of solution for the fractional nonlinear model of the low-pass electrical transmission lines via Atangana–Baleanu derivative operator. *Alex. Eng. J.* **59**(3), 1205–1214 (2020).

[15] Chou, D., Rehman, H. U., Amer, A. & Osman, M. Optical soliton dynamics of the conformable nonlinear evolution equation in Bose–Einstein condensates. *Rendiconti Lincei. Scienze Fisiche e Naturali* 1–12 (2024).

[16] Khater, M. M., Ghanbari, B., Nisar, K. S. & Kumar, D. Novel exact solutions of the fractional Bogoyavlensky–Konopelchenko equation involving the Atangana–Baleanu–Riemann derivative. *Alex. Eng. J.* **59**(5), 2957–2967 (2020).

[17] Ahmad, I., Ahmad, H., Thounthong, P., Chu, Y.-M. & Cesarano, C. Solution of multi-term time-fractional PDE models arising in mathematical biology and physics by local meshless method. *Symmetry* **12**(7), 1195 (2020).

[18] Chou, D., Ur Rehman, H., Amer, A. & Amer, A. New solitary wave solutions of generalized fractional Tzitzéica-type evolution equations using sardar sub-equation method. Opt. Quantum Electron. **55**(13), 1148 (2023).

[19] Shang, Y. The extended hyperbolic function method and exact solutions of the long-short wave resonance equations. *Chaos Solitons Fractals* **36**(3), 762–771 (2008).

[20] Elwakil, S. A., El-Labany, S., Zahran, M. & Sabry, R. Modified extended tanh-function method for solving nonlinear partial differential equations. *Phys. Lett. A* **299**(2–3), 179–188 (2002).

[21] Wang, M., Li, X. & Zhang, J. The (G’/G)-expansion method and travelling wave solutions of nonlinear evolution equations in mathematical physics. *Phys. Lett. A* **372**(4), 417–423 (2008).

[22] He, J. Variational iteration method for delay differential equations. *Commun. Nonlinear Sci. Numer. Simul.* **2**(4), 235–236 (1997).

[23] Vahidi, J. et al. New solitary wave solutions to the coupled Maccari’s system. *Results Phys.* **21**, 103801 (2021).

[24] Khater, M. M., Inc, M., Attia, R. A., Lu, D. & Almohsen, B. Abundant new computational wave solutions of the GM–DP–CH equation via two modified recent computational schemes. *J. Taibah Univ. Sci.* **14**(1), 1554–1562 (2020).

[25] Ablowitz, M. J., Kaup, D. J., Newell, A. C. & Segur, H. Method for solving the Sine–Gordon equation. *Phys. Rev. Lett.* **30**(25), 1262 (1973).

[26] Ablowitz, M. J. & Segur, H. Solitons and the inverse scattering transform. *SIAM* (1981).

[27] Gu, C., & Hu, H. Explicit solutions to the intrinsic generalization for the wave and Sine–Gordon equations. *Lett. Math. Phys.* **29**(1), 1–11 (1993).

[28] Gu, C., Hu, H. & Zhou, Z. Darboux Transformations in Integrable Systems: Theory and Their Applications to Geometry. Springer (2004).

[29] Wang, G., Yang, K., Gu, H., Guan, F. & Kara, A. A (2+ 1)-dimensional Sine–Gordon and Sinh–Gordon equations with symmetries and kink wave solutions. *Nucl. Phys. B* **953**, 114956 (2020).

[30] Akbar, M. A., Ali, N. H. M. & Hussain, J. Optical soliton solutions to the (2+1)-dimensional Chaffee–Infante equation and the dimensionless form of the Zakharov equation. *Adv. Differ. Equ.* **2019**, 1–18 (2019).

[31] Houwe, A. et al. Survey of third-and fourth-order dispersions including ellipticity angle in birefringent fibers on W-shaped soliton solutions and modulation instability analysis. *Eur. Phys. J. Plus* **136**, 1–27 (2021).

[32] Savaissou, N., Gambo, B., Rezazadeh, H., Bekir, A. & Doka, S. Y. Exact optical solitons to the perturbed nonlinear Schrödinger equation with dual-power law of nonlinearity. *Opt. Quant. Electron.* **52**, 1–16 (2020).

[33] Az-Zo’bi, E. A., Alzoubi, W. A., Akinyemi, L., Şenol, M. & Masaedeh, B. S. A variety of wave amplitudes for the conformable fractional (2+1)-dimensional ITO equation. *Mod. Phys. Lett. B* **35**(15), 2150254 (2021).

[34] Houwe, A. et al. Attitude of the modulation instability gain in oppositely directed coupler with the effects of the intrapulse Raman scattering and saturable function. *Results Phys.* **31**, 104851 (2021).

[35] Biswas, A. et al. Optical solitons for Lakshmanan–Porsezian–Daniel model by modified simple equation method. *Optik* **160**, 24–32 (2018).

[36] Yao, S.-W. et al. Dynamics of optical solitons in higher-order Sasa–Satsuma equation. *Results Phys.* **30**, 104825 (2021).

[37] Seadawy, A. R. Approximation solutions of derivative nonlinear Schrödinger equation with computational applications by variational method. *Eur. Phys. J. Plus* **130**, 1–10 (2015).

[38] Fujioka, J., Cortés, E., Pérez-Pascual, R., Rodríguez, R., Espinosa, A., & Malomed, B. A. Chaotic solitons in the quadratic-cubic nonlinear Schrödinger equation under nonlinearity management. *Chaos Interdiscip. J. Nonlinear Sci.* **21**(3) (2011). 10.1063/1.3629985 21974655

[39] Yokuş, A., Durur, H. & Duran, S. Simulation and refraction event of complex hyperbolic type solitary wave in plasma and optical fiber for the perturbed Chen–Lee–Liu equation. *Opt. Quant. Electron.* **53**, 1–17 (2021).

[40] Galaktionov, V. A. & Svirshchevskii, S. R. Exact Solutions and Invariant Subspaces of Nonlinear Partial Differential Equations in Mechanics and Physics. Chapman and Hall/CRC (2006).

[41] Tala-Tebue, E. & Seadawy, A. R. Construction of dispersive optical solutions of the resonant nonlinear Schrödinger equation using two different methods. *Mod. Phys. Lett. B* **32**(33), 1850407 (2018).

[42] Kaplan, M., Ünsal, Ö. & Bekir, A. Exact solutions of nonlinear Schrödinger equation by using symbolic computation. *Math. Methods Appl. Sci.* **39**(8), 2093–2099 (2016).

[43] Triki, H., Biswas, A., Moshokoa, S. P. & Belic, M. Optical solitons and conservation laws with quadratic–cubic nonlinearity. *Optik* **128**, 63–70 (2017).

[44] Li, C., Guo, Q. & Zhao, M. On the solutions of (2+ 1)-dimensional time-fractional Schrödinger equation. *Appl. Math. Lett.* **94**, 238–243 (2019).

[45] Khater, M. M., Seadawy, A. R. & Lu, D. Elliptic and solitary wave solutions for Bogoyavlenskii equations system, couple Boiti–Leon–Pempinelli equations system and time-fractional Cahn–Allen equation. *Results Phys.* **7**, 2325–2333 (2017).

[46] Rezazadeh, H. et al. Hyperbolic rational solutions to a variety of conformable fractional Boussinesq-like equations. *Nonlinear Eng.* **8**(1), 224–230 (2019).

[47] Choi, J., Kumar, D., Singh, J. & Swroop, R. Analytical techniques for system of time fractional nonlinear differential equations. *J. Korean Math. Soc.* **54**(4), 1209–1229 (2017).

[48] Tchier, F., Yusuf, A., Aliyu, A. I. & Inc, M. Soliton solutions and conservation laws for lossy nonlinear transmission line equation. *Superlattices Microstruct.* **107**, 320–336 (2017).

[49] Chou, D., Amer, A., Rehman, H. U. & Li, M.-L. Unravelling quiescent optical solitons: An exploration of the complex Ginzburg–Landau equation with nonlinear chromatic dispersion and self-phase modulation. *Nonlinear Eng.* **14**(1), 20240043 (2025).

[50] Younas, U. et al. Diverse exact solutions for modified nonlinear Schrödinger equation with conformable fractional derivative. *Results Phys.* **20**, 103766 (2021).

[51] Khalil, R., Al Horani, M., Yousef, A & Sababheh, M. A new definition of fractional derivative. *J. Comput. Appl. Math.* **264**, 65–70 (2014).

[52] Abdeljawad, T. On conformable fractional calculus. *J. Comput. Appl. Math.* **279**, 57–66 (2015).

[53] Mirzazadeh, M., Akinyemi, L., Şenol, M. & Hosseini, K. A variety of solitons to the sixth-order dispersive (3+ 1)-dimensional nonlinear time-fractional Schrödinger equation with cubic–quintic–septic nonlinearities. *Optik* **241**, 166318 (2021).

[54] Murad, M. A. S., Ismael, H. F., Hamasalh, F. K., Shah, N. A. & Eldin, S. M. Optical soliton solutions for time-fractional Ginzburg–Landau equation by a modified sub-equation method. *Results Phys.* **53**, 106950 (2023).

[55] Başhan, A. Solitary wave, undular-bore and wave-maker solutions of the cubic, quartic and quintic nonlinear generalized equal width (GEW) wave equation. *Eur. Phys. J. Plus* **138**(1), 53 (2023).

[56] Rehman, H. U. et al. Unraveling the (4+1)-dimensional Davey–Stewartson–Kadomtsev–Petviashvili equation: Exploring soliton solutions via multiple techniques. *Alex. Eng. J.* **90**, 17–23 (2024).

[57] Zhou, J. *Differential Transformation and Its Applications for Electrical Circuits Borneo Huazhong University Press* (Wuhan, China, 2010).

[58] Ibrahim, R. & Abdelaziz, M. S. Application of differential transform method with Adomian polynomial for solving RLC circuits problems and higher order differential equations. *Eng. Res. J. (Shoubra)* **51**(4), 89–95 (2022).

[59] Zahran, E. H., Umurzakhova, Z., Bekir, A., Myrzakulov, R. & Ibrahim, R. A. New diverse types of the soliton arising from the integrable Kuralay equations against its numerical solutions. *Eur. Phys. J. Plus* **139**(11), 1–18 (2024).

[60] Patel, Y. F. & Dhodiya, J. M. Applications of Differential Transform to Real World Problems. Chapman and Hall/CRC (2022).

[61] Aslan, E. C. & Inc, M. Soliton solutions of NLSE with quadratic–cubic nonlinearity and stability analysis. *Waves Random Complex Media* **27**(4), 594–601 (2017).

[62] Attia, R. A., Khater, M., El-Sayed Ahmed, A. & El-Shorbagy, M. Accurate sets of solitary solutions for the quadratic–cubic fractional nonlinear Schrödinger equation. *AIP Adv.* **11**(5) (2021).

